# Comparison of Fusion Rates between Glycerol-Preserved and Frozen Composite Allografts in Cervical Fusion

**DOI:** 10.1155/2014/960142

**Published:** 2014-10-28

**Authors:** Ian Rodway, Julie Gander

**Affiliations:** Beacon Orthopaedics & Sports Medicine, 500 E Business Way, Suite A, Sharonville, OH 45241, USA

## Abstract

*Background*. This retrospective, two cohort series study was designed to compare a room temperature, glycerol-preserved composite pinned bone allograft (G-CPBA) with the same graft type provided in a frozen state (F-CPBA) for use as a cervical interbody spacer in anterior cervical discectomy and fusion (ACDF). *Methods*. A comprehensive chart review was performed for 67 sequential patients that received either a F-CPBA or a G-CPBA and had at least one-year follow-up. Twenty-eight patients had received G-CPBA grafts and 37 patients had received F-CPBA grafts. Two additional 2-level patients had received one of each type of grafts. *Results*. At 3 months, 45.3% (29 of 64) of glycerol-preserved and 41.4% (29 of 70) of frozen allografts, respectively, were considered to be fused radiographically. At 12 months, 100% of both treatment groups (41 glycerol-preserved and 45 frozen) were considered fused. Fusion rates for G-CPBA were statistically similar to F-CPBA at both 3 and 12 months (*P* = 0.6535 and >0.999, resp.). There were no allograft related complications in either treatment group. *Conclusions*. 100% fusion rates were attained by both treatment groups at 12 months and were similar at short-term follow-up for all comparable levels. *Level of Evidence*. Level of evidence is III.

## 1. Introduction

Cervical spondylosis is a problem commonly resulting from degeneration of the cervical intervertebral discs. While many cases can be treated nonsurgically, some conditions deteriorate to cervical radiculopathy or myelopathy requiring surgical intervention, such as anterior cervical discectomy and fusion (ACDF). These procedures, with up to 500,000 annually [1], may employ synthetics, autologous bone, or allograft bone as interbody spacers. While autologous bone grafts were long considered a gold standard for use in fusion procedures, the disadvantages include donor site pain and morbidity, increased operative time, and reliance on the quality of the often ill, patient's own bone. Given these issues and the advent of readily available, precisely shaped, and sterile allograft bone, there has been a shift towards this surgical option [2]. In particular, composite allografts consisting of cortical side plates for strength and a cancellous core for enhanced fusion have been successfully used in ACDF cases [1, 3]. Allograft bone has traditionally been either preserved and stored at room temperature following lyophilization or provided frozen. However, for lyophilized bone, the process of freeze-drying can alter biomechanical properties [4, 5], and rehydration of the grafts prior to implantation can be time-consuming and may not fully restore native properties [6]. While frozen allografts may retain biomechanical properties, the need for low temperature shipment and subsequent storage can be challenging and costly.

In addressing the shortcomings of both freeze-dried and low temperature options, a glycerol-based preservation method has been introduced [7–10] to allow non-freeze-dried preservation of bone at room temperature and yielding a ready-to-use graft. Of concern to this approach, the use of glycerol in spinal grafts has been brought into question due to a report [11] of several animal deaths following implantation of a large quantity of bone void filler with glycerol carrier. However, a follow-up study [12] indicated that implantation of only clinically relevant quantities of glycerol-based bone void filler was nontoxic. Furthermore, several clinical studies indicate that glycerol-based bone void fillers, which include up to 70% glycerol, are effective in spinal fusion procedures [13–15]. However, there is no known published report of the use of a glycerol-preserved interbody spacer. Here, we report fusion rates of a glycerol-preserved composite bone graft as an interbody spacer in ACDF procedures using an identically constructed frozen allograft as control.

## 2. Materials and Methods

### 2.1. Design and Objectives

This study compares two consecutive cohort series undergoing ACDF surgeries using a composite allograft interbody spacer. The composite nature of this allograft is due to the lateral sides of the graft consisting of cortical planks to provide strength while the center contains a cancellous block to enhance fusion. The segments are assembled and held in place using cortical pins. One study arm consisted of allografts stored and provided frozen until the time of surgery and the other study arm included allografts that were glycerol-preserved and provided at room temperature. The patients were chosen consecutively based on surgery date without regard for surgery levels, risk factors, or other conditions. The study included 67 total C3–C7 ACDF patients, at surgery levels of 1, 2, and 3, and a single 4-level case. Sixty-five patients exclusively received either glycerol-preserved or frozen allografts. Two additional patients received 1 of each type of allografts. The endpoints were assessment of fusion at 3 and 12 months postoperatively and any adverse events or other significant observations. Fusion rates as a function of graft type and number of patients as well as levels of surgery were calculated. This retrospective cohort comparison study has a level of evidence of III.

### 2.2. Patient Population

All patients were treated by the same clinician (I.R.). The use of glycerol-preserved composite allografts for ACDF commenced in this practice early in 2011 and replaced the frozen graft option. The two cohorts were thus chronologically sequential. The study included 67 patients who had a sufficient follow-up period and were between the ages of 33 and 74. Clinical evaluations were performed on 37 consecutive patients (surgery date: from January 2010 to March 2011) with frozen allografts and 28 consecutive patients (surgery date: from February 2011 to July 2011) with glycerol-preserved allografts. Additionally, two patients overlapped in both the glycerol-preserved and frozen groups and received one of each of the two treatment grafts in 2-level procedures. Patients were only excluded from analysis when they did not return for sufficient follow-up. High risk patients (cigarette smokers, diabetics, those with rheumatoid arthritis, those with thyroid disorders, and those on chronic steroid therapy) were not excluded from the study.

### 2.3. Documentation

Write-ups of surgical experience were used retrospectively for evaluation of several conditions. Pain was evaluated by standard documentation assessment and notation of issues. Manual muscle testing was performed for the bilateral upper extremities and graded on a 5-point scale. Assessment was made in regards to intact sensation to light touch in all dermatomes, and description was noted if the patient responded negatively. Presence of Hoffman's sign was noted as positive or negative. X-rays were taken at 3, 6, and 12 months to evaluate fusion. The write-ups of patients receiving G-CPBA were compared with a similar cohort of patients using F-CPBA who were evaluated with identical documentation sheets.

### 2.4. Surgical Technique

Instrumented fusion with similar instruments/techniques was used for all procedures. Prior to use, the respective allografts (VG2 Cervical, LifeNet Health, Virginia Beach, VA), frozen or glycerol-preserved (Preservon, LifeNet Health, Virginia Beach, VA), were prepared as per manufacturer's instructions. The frozen allografts were thawed for at least 30 seconds in room temperature sterile saline prior to implantation. The glycerol-preserved grafts were reported by the manufacturer to contain no more than 5% glycerol as packaged and prior to presurgical rinse. The glycerol-preserved grafts were soaked in sterile saline for 30 seconds prior to implantation.

### 2.5. Postoperative Care

Patients were seen and examined, and AP and lateral radiographs were performed, at 2 weeks, 6 weeks, 3 months, 6 months, and 1 year.

## 3. Statistical Methods

Statistical significance (*P* ≤ 0.05) was calculated for fusion rates between the frozen and glycerol-preserved treatment groups using Student's *t*-test and Microsoft Excel (*Microsoft, Redmond, WA*). Statistical significance was evaluated for all levels of surgery at 3 and 12 months follow-up.

## 4. Results

**Figure 1:**
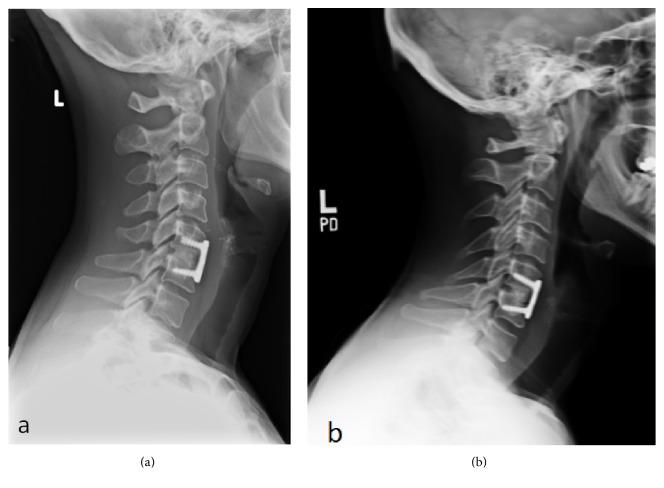
Six months postoperative X-ray images showing fusion for 1-level ACDF treated using (a) frozen allograft and (b) glycerol-preserved allograft.

**Figure 2:**
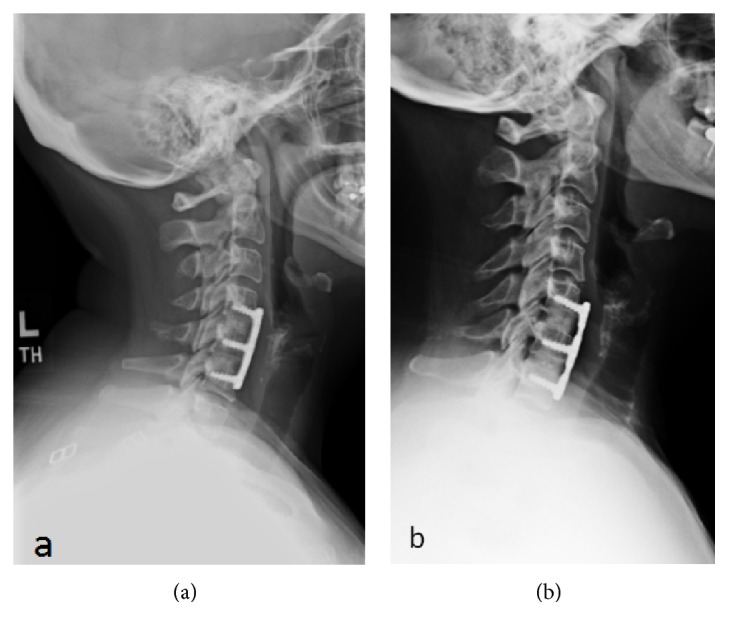
Six months postoperative X-ray images showing fusion for 2-level ACDF treated using (a) frozen allografts and (b) glycerol-preserved allografts.

Fusion data was collected for each treatment group and according to level of surgery (Tables 1 and 2). When assessing fusion across total levels of surgery, the two patients receiving one of each type of allografts were considered to be level 2 surgeries counting as one-half of a patient for each allograft type. At 3 months, 37.9% (11 of 29) of patients in the glycerol-preserved group and 42.1% (16 of 38) of patients in the frozen group were considered to be fused radiographically. At 12 months, 100% of patients in both treatment groups (17.5 glycerol-preserved and 24.5 frozen) were considered fused. Fusion rates for patients in the glycerol-preserved treatment group were statistically similar to patients in the frozen allograft group at both 3 and 12 months (*P* = 0.7343 and >0.9999). Representative X-rays of grafts considered fused in either 1- or 2-level cases are shown in Figures 1 and 2, respectively. There were no allograft related complications in either treatment group.

At 3 months, 45.3% (29 of 64) of glycerol-preserved levels and 41.4% (29 of 70) of frozen levels were considered to be fused radiographically. At 12 months, 100% of both treatment groups (41 glycerol-preserved and 45 frozen) were considered fused. Fusion rates for glycerol-preserved allografts were statistically similar to frozen allografts at both 3 and 12 months (*P* = 0.6535 and >0.999, resp.). At 3 months, 1-level and 2-level treatments showed a combined fusion rate of 38.9% (14 of 36) for glycerol-preserved allografts and 44.2% (23 of 52) for frozen allografts. These were statistically similar (*P* = 0.6217). The more complex 3-level treatments showed fusion rates of 45.8% (11 of 24) for glycerol-preserved allografts and 33.3% (6 of 18) for frozen allografts. These were also statistically similar (*P* = 0.4235). While there were not any 4-level frozen allografts patients for comparison, the single 4-level glycerol-preserved allograft patient showed 100% fusion at 3 months. All tobacco users that reached 12 months assessment, 4 for frozen allografts and 3 for glycerol-preserved allografts, demonstrated fusion.

## 5. Discussion

While freeze-dried and frozen allografts can have limitations with regards to altered biomechanics after processing and inconvenient preparation for surgical use, glycerol-preserved allografts appear to offer a viable alternative based on the results of this study. Fusion rates for the patients in the glycerol-preserved group remained comparable to those in the frozen group at the 3- and 12-month follow-up periods. Additionally, the safety concern of using glycerol-preserved allografts appears to be a nonissue as there were neither signs of toxicity nor other clinical observations related to the glycerol-preserved group. Interestingly, there were two patients that underwent 2-level procedures that had one of each of the two implant types. Both patients exhibited fusion at both levels at three months.

Although patients in the frozen allograft treatment group had a slightly higher fusion rate (42.1 versus 37.9%) at 3 months follow-up, the glycerol-preserved treatment group had a slightly higher fusion rate when assessed by number of levels (45.3 versus 41.4%), and both analyses showed no statistical difference between frozen and glycerol-preserved groups (*P* = 0.7343 and 0.6535, resp.). These data suggest that there are no short-term differences in the outcomes of the two treatments. At the end point of the study, 12 months follow-up, both allograft treatment groups exhibited 100% fusion. In comparing tobacco users and nonusers, fusion rates differ largely between the two subgroups with a fusion rate of 44.6% for nonusers versus 18.2% for tobacco users at 3 months postoperatively. This result appears to be in line with current knowledge about the detrimental effects of nicotine on the healing process [16–18]. However, the difference between tobacco users and nonusers diminished by 12 months postoperatively, as both groups exhibited 100% fusion.

Study limitations include the use of only one surgical practice and clinician, although this has the advantage of consistent surgical technique. Another limitation is the chronologically sequential, nonparallel design of the study where, with minor exception, the frozen graft series occurred prior to the glycerol-preserved graft series. Also, the retrospective nature of the study limited data that could be collected; thus, standard measurements such as visual analog score for pain and Oswestry or Neck Disability Indices scores were unavailable. However, the use of one implanting physician, consistent allograft composition, and instrumentation should have provided consistency of treatment and an accurate comparison of the test versus control arms of the study.

One hundred percent fusion rates were attained by both the traditional frozen and the novel glycerol-preserved allograft groups. Fusion rates were also similar at short-term follow-ups, suggesting that there is not a significant difference in clinical outcomes with either treatment. While not generalizable, these results are encouraging and support the use of glycerol-preserved allografts for ACDF surgery.

## Figures and Tables

**Table 1:** Fusion rates by numbers of patients^*^.

	Frozen				Glycerol-preserved				Frozen versus glycerol statistically similar?
	Total patients	Fused grafts	Not fused	% of fused patients	Total patients	Fused grafts	Not fused	% of fused patients	
Three months	38	16	22	42.1%	29	11	18	37.9%	Yes (*P* = 0.7343)
12 months	24.5	24.5	0	100%	17.5	17.5	0	100%	Yes (*P* > 0.999)

^*^Note that 2 patients had both glycerol-preserved and frozen grafts, 1 level for each type. The results from those patients were counted as 1/2 patients in each group.

**Table 2:** Fusion rates by numbers of levels^*^.

		Frozen				Glycerol-preserved				Frozen versus glycerol statistically similar?
		Total levels	Fused grafts	Not fused	% of fused levels	Total levels	Fused grafts	Not fused	% of fused levels	
Three months	Total	70	29	41	41.4%	64	29	35	45.3%	Yes (*P* = 0.6535)
	1 level	12	6	6	50.0%	4	1	3	25.0%	Yes (*P* = 0.4283)
	2 levels	40	17	23	42.5%	32	13	19	40.6%	Yes (*P* = 0.8748)
	3 levels	18	6	12	33.3%	24	11	13	45.8%	Yes (*P* = 0.4235)
	4 levels	0	0	0	—	4	4	0	100%	—

12 months	Total	45	45	0	100.0%	41	41	0	100%	Yes (*P* > 0.999)
	1 level	8	8	0	100.0%	2	2	0	100%	Yes (*P* > 0.999)
	2 levels	25	25	0	100.0%	17	17	0	100%	Yes (*P* > 0.999)
	3 levels	12	12	0	100.0%	18	18	0	100%	Yes (*P* > 0.999)
	4 levels	0	0	0	—	4	4	0	100%	—

^*^Note that 2-level patients had both glycerol-preserved and frozen grafts. The results from those patients were counted as 1 level in each 2-level treated group.
